# High Temperature Ceramic Materials

**DOI:** 10.3390/ma14082031

**Published:** 2021-04-17

**Authors:** Shaowei Zhang

**Affiliations:** College of Engineering, Mathematics and Physical Sciences, University of Exeter, Stocker Rd, Exeter EX4 4PY, UK; s.zhang@exeter.ac.uk

Thanks to their superior physiochemical properties such as high melting point, excellent mechanical properties, good thermal properties, and great corrosion/erosion resistance, high temperature ceramic materials (HTCM) find applications in a broad range of demanding areas or industrial sectors, e.g., in metallurgy, glass, cement, aerospace, nuclear, and power generation, contributing substantially to our sustainable society.

HTCM refers mainly to high melting point oxides, carbides, nitrides, and borides. In the past two decades, and in particular, in recent years, significant amounts of research work have been carried out aiming to improve their properties/performance; enhance their service lives; and to deal with several critical issues related to production cost, sustainability, and eco-friendliness. Some of these topical areas include: (1) exploration of new system of ceramics, (2) low temperature synthesis of high quality ceramic particles, (3) graphene and/or carbon nanotube reinforced ceramic composites, (4) “green” low-carbon refractory composites, (5) energy-saving lightweight ceramics, (6) novel catalytic synthesis of ceramic materials, (7) high/low thermal conductivity ceramics, (8) self-healing ceramic composites, (9) ultra-high temperature ceramics, (10) additive manufacturing of ceramics, (11) directionally solidified eutectic ceramics, (12) functional ceramic coatings, (13) plasma/cold/flash sintering, (14) recycling of waste materials, (15) new testing techniques, and (16) ceramic materials modelling/simulation.

This Special Issue, “High Temperature Ceramic Materials”, released by Materials, is dedicated to original research articles from the ceramics/refractories communities, presenting some of the latest work in a few topical areas listed above. In total, 15 good quality articles have been included [1,2,3,4,5,6,7,8,9,10,11,12,13,14,15], the interesting points from each of which are briefly highlighted as follows.

In ceramic engineering, the preparation and use of high-quality raw materials is critical to fabricating bulk components with guaranteed properties/performance/service life. Given the drawbacks of conventional synthesis techniques (e.g., the requirement of high synthesis temperature), it is necessary to develop other novel techniques to synthesise high-quality ceramic powder at much milder conditions. In response to this, a group from the UK [2,5] have developed a low temperature molten salt synthesis (MSS) technique and successfully synthesised fine particles of aluminium boron carbide and MoAlB, two promising high temperature ceramic materials, at much lower temperatures. Interestingly, the MSS technique also can be extended or modified to synthesise other types of high melting point ceramic materials with novel morphologies for both structural and functional applications. As demonstrated by Liu et al. [7], by combining it with microwave heating, TiB_2_ microplatelets can be synthesised at a lowered temperature and/or in a shorter time. On the other hand, by combining it with chemical vapour deposition, 1-D necklace-like SiC/SiO_2_ heterojunctions can be prepared without using any catalyst [14].

Given the importance of shape-forming and subsequent sintering/densification in the preparation of bulk ceramics, Jin et al. [10] have investigated and compared sintering/densification behaviours of MgO green samples resulting, respectively, from a conventional air compaction and a novel vacuum compaction. They have found that air pockets entrapped in the green samples play an important role in their densification and defect formation.

Compared with their dense counterparts, porous ceramics exhibit several advantages, in particular, lower thermal conductivity and lighter weight. In the work from Li et al. [11], porous SiC ceramics with high flexural strengths at both room and high temperatures have been successfully prepared via hot pressing assisted with liquid sintering agents. Additionally, another group [9] have prepared porous α-Sialon ceramics using an improved unidirectional freeze casting method, in which a gradient porous structure has been formed by taking advantage of the decreased solidification velocity in the freezing direction.

Apart from monolithic ceramics, several types of ceramic matrix composites for high temperature applications have been investigated. As illustrated in Ref. [13], carbon content in an MgO–carbon refractory composite used for steel making shows great effects on its mechanical strength, oxidation resistance, and slag corrosion resistance. The data presented could be beneficial to the further development of the so-called “low carbon carbon-containing refractories” for clean steel making. In another work [1], the promising effect of ZrC nanoparticle incorporation on the microstructure and mechanical properties of carbon fibre-reinforced SiC composites at both room and elevated temperatures has been revealed. On the other hand, by using the newly designed atmosphere-controllable “coupling thermal–mechanical material test device”, Yue et al. [4] have examined the residual mechanical strength of an ultra-high temperature ZrB_2_–SiC–graphite composite under complex thermal and mechanical conditions, and evaluated its thermal shock resistance. In addition, by using a high-speed infrared camera to monitor the surface temperature of a model ceramic matrix composite subjected to different levels of uniaxial tensile stress, Kim [6] has managed to establish the relationship between the surface temperature change and the mechanical testing data. This, along with microstructural characterisation, can be used to identify the failure mode and failure mechanism of a ceramic matrix composite.

During service at elevated temperatures, ceramic materials interact with their service environments, which dominates their overall performance and service lives. Considering this, a group from China have investigated interactions between high melting point oxide-based crucible materials and molten superalloys under vacuum induction melting conditions [12,15]. The data would be useful for future crucible material selection and design in this and relevant areas. In addition to these, in another work [8], an innovative approach based on big data mining methods has been proposed to simulate and predict the electrical conductivity of a molten slag, an important parameter affecting metallurgical processes, as well as the degradation of refractory lining materials.

Last, but not least, an additional challenging issue concerning the high temperature ceramic industry is regarding the recycling of industrial wastes (including its own wastes). Recovered materials are now commonly regarded as invaluable commodities. In this regard, Liu et al. [3] have proposed to use the slag from copper and stainless steel making to fabricate black ceramic tiles, opening up new avenues for the low-cost and environmentally friendly production of high-quality black ceramic tiles, as well as value-added utilization of the two types of wastes.
